# The Hospital Patient's Point of View

**Published:** 1913-05-31

**Authors:** 


### THE HOSPITAL PATIENT'S POINT OF VIEW.

It is not often, doubtless, that a hospital entertains unawares among its patients a critic as acute, as observant, as open-minded, and as good-tempered as the layman whose instructive comments from the patient's point of view we print on pages 281, 282. It is just because of the qualities of sweet reasonableness and of able advocacy so conspicuous in his essay that some analysis of his criticisms is essential. For it would be fair neither to our voluntary hospitals to allow them to pass unchallenged altogether, nor to our contributor to pooh-pooh him as a disgruntled faddist.

It will be noticed that in the main the nursing home in which his first experience of operative surgery occurred receives ungrudging admiration. The cobble-paved square outside his window does, it is true, come in for malediction on account of the noise created in it by passing traffic; but this is really the local authority's affair, not that of the nursing home proprietor, except in so far as the latter may have been responsible for selecting the site of the home. In the amusing account, too, of the ordeal of entry into the operating theatre there is a gibe at the formality of introducing the patient to the anæsthetist and to the surgeon's assistant. We may concede the essential futility of this conventional usage; probably no one was so fully aware of it as the other parties to the transaction. Really this is not the fault of the doctors, but of the public at large, 99 per cent. of whom would feel mortally offended were this useless ceremonial omitted. The third criticism is directed against the practice of anæsthetising the patient in the actual operating theatre, and here our layman is on surer ground. The probable explanation is that no ante-room could be arranged when the building was adapted to the purpose of a nursing home. If it was actually built for one, certainly there was a defect in its design in this particular.

Proceeding to his later experiences in the pay wing of a general hospital, the patient has several pertinent comments to offer. To begin with, he seems to have been left somewhat severely alone, perhaps because the available nursing staff was attending to those actually in need of constant care. Then a nurse is stated to have offered him a drink of medicine, with "the direst results." If this means that the drug was an aperient the complaint cannot be serious, for the precaution is an essential one. Proceeding, there is mention of a hypodermic injection before the operation, though it is not disclosed of what it consisted. If it was one of the now fashionable narcotic alkaloids, the object was to spare the patient discomfort, and this object seems to have been duly attained.

Some of his further grievances have, however, more substance. Distressing flatulence after nephrotomy is unfortunately not uncommon and not always easy to relieve; but inadequacy of the nursing arrangements in the home does seem to have obtained, and avoidable noise is another defect which should not exist in a well conducted institution of any sort. Imperfection in respect of some of the ward utensils has also to be admitted if the account given is accurate. And it is further evident that some of the nurses had not completed their training, in itself not a serious matter except they were clearly incompetent.

Lastly, our layman complains that he was only "a case," not a human being, to those in charge of him. In an institution such as a general hospital it is difficult to see how this is to be always provided against. To cultivate intimacy with every patient is plainly impossible for nurses or medical staff in every large ward, nor would it be often desirable. As a matter of fact, patients who, by reason of some obstinate disease, remain many weeks or months in hospital wards often do manage to impress the sense of their personality upon their attendants, and do receive extra "notice" in consequence; but the process naturally takes some time, and is much less easily and naturally brought about in a ward. If, as we gather, it was in the nursing home of the hospital the case is different. The fact, as our contributor notes, that that patient is the most esteemed in hospital who makes the least fuss and gives the least trouble is only what is to be expected while human nature remains what it always has been. This criticism deserves to be weighed and noted, but even more serious are those about avoidable noise and poor attendance. Still, as long as the patient's point of view is put with the moderation and good humour of our anonymous contributor we do not think institutional managers will feel inclined to resent it.

